# Central kisspeptin injection enhances food consumption in broiler chickens: role of opioidergic and dopaminergic receptors

**DOI:** 10.1016/j.psj.2025.106007

**Published:** 2025-10-23

**Authors:** Hamed Zarei, Mohammadrasol Radmehr, Alireza Mohtasham, Mehran Mehdipour, Keyvan Hasani

**Affiliations:** a Department of Biology, CT.C., Islamic Azad University, Tehran, Iran; b Department of Basic Sciences of Veterinary Medicine, Islamic Azad University, Garmsar Branch, Garmsar, Iran

**Keywords:** Kisspeptin, Dopaminergic, Opioidergic, Food intake, Intracerebroventricular injection

## Abstract

Kisspeptin, acting as both a neuropeptide and a hormone, known for its pivotal role in reproductive function and energy homeostasis, exerts multifaceted effects on central nervous system pathways involved in appetite regulation. Therefore, this study aimed to examine the impact of intracerebroventricular (ICV) infusion of kisspeptin on feeding behavior in neonatal broilers and to elucidate its interaction with the opioidergic and dopaminergic systems within the hypothalamic circuits governing appetite. Across seven separate experiments, broilers were systematically allocated into four different treatment groups for each experiment. In the initial experiment, neonatal chickens received ICV injections of saline or kisspeptin at escalating doses (0.25, 0.5, and 1 nmol). Subsequent experiments assessed the interplay between kisspeptin (1 nmol) and opioidergic modulators: β-FNA (mu opioid receptor antagonist), nor-BNI (kappa opioid receptor antagonist), and NTI (delta opioid receptor antagonist), as well as dopaminergic agents: l-DOPA (Dopamine precursor), SCH 23390 (D1 receptor antagonist), and AMI-193 (D2 receptor antagonist). Treatments included individual injections and simultaneous co-administrations of kisspeptin with these pharmacological agents. Feed consumption was quantitatively measured for 120 min post-infusion. Kisspeptin administration elicited a dose-dependent augmentation in feed consumption (*P* < 0.05). Co-infusion of l-DOPA+ kisspeptin inhibited the orexigenic effect induced by kisspeptin (*P* < 0.05). However, simultaneous infusion of β-FNA and SCH 23390 with kisspeptin markedly increased kisspeptin-stimulated hyperphagia (*P* < 0.05). The data indicate that kisspeptin promotes hyperphagia in neonatal broiler-type chickens, and the hyperphagic response is mediated through mu-opioid and D1-dopaminergic receptor pathways.

## Introduction

The regulation of appetite in birds involves homeostatic mechanisms distinct from those in mammals, reflecting unique neurobiological adaptations (Tachibana and Tsutsui, 2016). Feeding behavior is influenced by nutrient composition and the integrated actions of central neurotransmitters that either inhibit or stimulate hypothalamic feeding circuits (Rahmani et al., 2021). The hypothalamus functions as a critical node in the control of this regulation by controlling various peptides and neurotransmitters (Yousefvand and Hamidi, 2022). This central control of appetite assumes particular importance in broilers, where growth rate is closely linked to food intake, highlighting the need for precise appetite regulation in poultry production (Korver, 2023; Richards, 2003).

Among key hypothalamic peptides modulating appetite, kisspeptin—a neuropeptide and hormone encoded by the KISS1 gene—plays an essential role in coordinating reproductive function and energy homeostasis across vertebrate species (Xie et al., 2022). The expression of this factor is prominent in hypothalamic nuclei associated with metabolic control, including the arcuate (ARC) and paraventricular nuclei (PVN) (Prashar et al., 2023). Kisspeptin interacts with neuropeptides such as neuropeptide Y (NPY) and pro-opiomelanocortin (POMC), integrating reproductive and metabolic signaling (Azizi et al., 2021; Kord et al., 2022). While central kisspeptin administration suppresses feeding in some mammalian models (Cázarez‐Márquez et al., 2021), it stimulates meal consumption in chickens (Mahdavi et al., 2024). This marked species difference in the feeding response to kisspeptin between mammals and birds underscores the need for species-specific investigations. Although the canonical mammalian KISS1 and KISS1R genes are absent in birds (Pasquier et al., 2014), empirical physiological evidence demonstrates that exogenous kisspeptin administration exerts potent, receptor-mediated effects on food intake and reproduction in avian species, indicating the presence of a functional binding and signaling system (Khan et al., 2009; Wu et al., 2013; Kord et al., 2022). These multifaceted roles position kisspeptin as a critical modulator of meal intake, warranting detailed investigation of its mechanisms and interactions with other neuromodulatory systems in broiler chickens.

In addition to hypothalamic peptides like kisspeptin, the opioidergic system is a crucial modulatory pathway involved in both homeostatic and reward-related aspects of feeding (Mahdavi et al., 2023a). In birds, μ-opioid receptor activation typically suppresses food intake, whereas δ- and κ-receptor stimulation promotes it (Bungo et al., 2005). The μ-opioid receptor often mediates the feeding effects of other hormones, highlighting its integrative role (Zendehdel et al., 2016). Similarly, the dopaminergic system is fundamental to feeding and metabolic regulation. Dopamine acts through D1 and D2 receptor subtypes, which are prominently expressed in brain regions crucial for appetite control and mediate the effects of various neurotransmitters on feeding behavior (Klein et al., 2019; Mahdavi et al., 2024; Zarei et al., 2025b).

While the individual roles of kisspeptin, opioidergic, and dopaminergic systems in food intake regulation are well documented, emerging evidence points to complex interactions among these pathways that remain largely unexplored in birds. No previous study has specifically investigated the interaction between central kisspeptin and opioidergic/dopaminergic systems on feeding behavior in broiler chickens. Mammalian studies suggest substantial crosstalk: beta-endorphin modulates kisspeptin neurons in a photoperiod-dependent manner, influencing pulsatile gonadotropin-releasing hormone (GnRH) release (Hellier et al., 2023); neurokinin 3 and kappa-opioid receptors respectively excite and inhibit neurons characterized by the co-expression of neurokinin B, dynorphin, and kisspeptin (Ruka et al., 2013). Dopamine further modulates kisspeptin signaling by suppressing hypothalamic kisspeptin neurons, thereby influencing ghrelin secretion and reproductive axis regulation (Khosravi et al., 2018; Sadeghzadeh et al., 2018), while kisspeptin restrains prolactin release through inhibition of dopaminergic neurons in an estradiol-dependent manner (Szawka et al., 2010). Despite these insights, the combined effects of these neuromodulatory systems on feeding remain undefined. Accordingly, this investigation focuses on understanding the role of central kisspeptin infusion in modulating meal consumption in broiler chickens, emphasizing the mediating roles of opioidergic and dopaminergic receptor systems.

## Materials and methods

### Animal procurement and husbandry

A total of 308 one-day-old broiler-type chicks (Ross-308) were procured from a certified local hatchery (Mahan Company, Tehran, Iran). Upon arrival, the chicks were housed communally under a controlled environment featuring consistent photoperiods and a temperature maintained at 31 ± 2°C for an initial two-day acclimation period. Following this adaptation phase, individuals were randomly allocated to separate cages to standardize environmental exposure. Chicks were individually marked and randomly allocated into experimental groups (*n* = 11 per group for each of the 7 experiments, see Table 1 for group details). Throughout the experimental timeline, the chicks were fed ad libitum a nutritionally balanced starter diet formulated specifically to support optimal early growth, as detailed in Table 2. At postnatal day five, chicks underwent pharmacological treatments administered via intracerebroventricular (ICV) injection (Saffar et al., 2025). Prior to ICV infusion, all subjects were fasted for three hours, with unrestricted access to water to minimize confounding effects on food intake, following established protocols (Zarei et al., 2025a). All animal handling, housing, and experimental procedures complied strictly with the ethical standards outlined by the Iranian national regulations on animal care and use, as well as the guidelines of the National Institutes of Health (USA) for laboratory animal welfare. Ethical approval was obtained from the Animal Ethics Committee of the Central Tehran Branch of Islamic Azad University, Tehran, Iran (approval number IR.IAU.CTB.REC.1403.098).

**Table 1:** Sequence of pharmacological interventions in experimental groups.

Experiments	Groups			
	A	B	C	D
**1**	CS*	Kisspeptin (0.25 nmol)	Kisspeptin (0.5 nmol)	Kisspeptin (1 nmol)
**2**	CS	β-FNA (5 μg)	Kisspeptin (1 nmol)	β-FNA + Kisspeptin (5 μg) + (1 nmol)
**3**	CS	nor-BNI (5 μg)	Kisspeptin (1 nmol)	nor-BNI + Kisspeptin (5 μg) + (1 nmol)
**4**	CS	NTI (5 μg)	Kisspeptin (1 nmol)	NTI + Kisspeptin (5 μg) + (1 nmol)
**5**	CS	L-DOPA (125 nmol)	Kisspeptin (1 nmol)	L-DOPA + Kisspeptin (125 nmol) + (1 nmol)
**6**	CS	SCH 23390 (5 nmol)	Kisspeptin (1 nmol)	SCH 23390 + Kisspeptin (5 nmol) + (1 nmol)
**7**	CS	AMI-193 (5 nmol)	Kisspeptin (1 nmol)	AMI-193 + Kisspeptin (5 nmol) + (1 nmol)

Groups A, B, C, and D represent the treatment allocations for each independent experiment (e.g., control (A) and different doses of kisspeptin (B–D) in Experiment 1; and control (A), single-agent (B), kisspeptin (1 nmol; C), and co-administration treatment (D) in subsequent experiments.

CS refers to the control solution containing Evan’s Blue dye; β-FNA (β-funaltrexamine, antagonist specific to the μ receptor); nor-BNI (norbinaltorphimine, antagonist specific to the κ receptor); NTI (naltrindole, antagonist specific to the δ receptor); l-DOPA (Dopamine precursor); SCH 23390 (antagonist specific to the D1 receptor); AMI-193 (antagonist specific to the D2 receptor**).**

**Table 2:** Assessment of the components and nutrient profile of the diet used in the experiment.

Ingredient (%)		Nutrient analysis	
Corn	52.85	ME, kcal/g	2850
Soybean meal, 48 % CP	31.57	Crude protein (%)	21
Wheat	5	Linoleic acid (%)	1.69
Gluten meal, 61 % CP	2.50	Crude fiber (%)	3.55
Wheat bran	2.47	Calcium (%)	1
Di-calcium phosphate	1.92	Available phosphorus (%)	0. 5
Oyster shell	1.23	Sodium (%)	0.15
Soybean oil	1.00	Potassium (%)	0.96
Mineral premix	0.25	Chlorine (%)	0.17
Vitamin premix	0.25	Choline (%)	1.30
Sodium bicarbonate	0.21	Arginine (%)	1.14
Sodium chloride	0.20	Isoleucine (%)	0.73
Acidifier	0.15	Lysine (%)	1.21
DL-Methionine	0.10	Methionine (%)	0.49
Toxin binder	0.10	Methionine + cystine (%)	0.83
L-Lysine HCl	0.05	Threonine (%)	0.70
Vitamin D_3_	0.1	Tryptophan (%)	0.20
Multi enzyme	0.05	Valine (%)	0.78

Metabolisable energy (ME) and crude protein (CP) are expressed per kilogram of diet. The mineral supplement comprises 35.2 g of manganese sourced from MnSO₄∙H₂O; 22 g of iron derived from FeSO₄∙H₂O; 35.2 g of zinc obtained from ZnO; 4.4 g of copper from CuSO₄∙5H₂O; 0.68 g of iodine provided as ethylenediamine dihydroiodide; and 0.12 g of selenium from Na₂SeO₃. The vitamin supplement includes 1.188 g of retinyl acetate, 0.033 g of dl-α-tocopheryl acetate, 8.84 g of tocopherol, 1.32 g of menadione, 0.88 g of thiamine, 2.64 g of riboflavin, 13.2 g of nicotinic acid, 4.4 g of pantothenic acid, 1.76 g of pyridoxine, 0.022 g of biotin, 0.36 g of folic acid, and 1500 mg of choline chloride.

### Drugs

All pharmacological compounds were sourced from Sigma-Aldrich (St. Louis, MO, USA). The agents included kisspeptin, β-funaltrexamine (β-FNA; μ opioid receptor antagonist, 5 μg**)**, norbinaltorphimine (nor-BNI; κ opioid receptor antagonist, 5 μg), naltrindole (NTI; δ opioid receptor antagonist, 5 μg), l-DOPA (dopamine precursor, 125 nmol), SCH39166 (D1 receptor antagonist, 5 nmol), AMI-193 (D2 receptor antagonist, 5 nmol), along with Evans Blue. Stock solutions were prepared in absolute dimethyl sulfoxide (DMSO) and subsequently diluted with 0.85 % isotonic saline containing Evans blue dye, maintaining a dilution ratio of 1:250, which yielded a final DMSO concentration of 0.4 %. This concentration was expressly chosen based on previous evidence confirming negligible cytotoxic effects (Blevins et al., 2002; Ebrahimnejad et al., 2025; Mahdavi et al., 2023b). The vehicle control consisted of saline supplemented with 0.4 % DMSO and Evans blue.

### Intracerebroventricular injection and feed intake

Experimental sessions began by recording the body weight of each chick to allow subsequent normalization of feed intake relative to body mass. Intracerebroventricular infusions were performed without anesthesia, utilizing a Hamilton microsyringe (Switzerland). The stereotaxic injection technique employed was adapted from the methodology described by Furuse et al. (1997), incorporating an apparatus designed to orient the broiler's head at a constant 45-degree inclination, thereby ensuring accurate alignment of the calvarium and facilitating reproducible targeting of the ventricle. A transparent plastic plate with a pre-drilled aperture was positioned over the right lateral ventricle landmark, through which the syringe needle was inserted to a depth of 4 mm. This injection technique proved to be physiologically benign, eliciting no stress response in the chickens (Davis et al., 1979). All groups received an infusion of 10 μL at a constant volume, encompassing controls and pharmacological treatments (detailed in Table 1). Dosages were selected based on previously validated studies to ensure efficacy while avoiding toxicity (Kord et al., 2022; Zendehdel et al., 2016). We administered selective antagonists and modulators of μ-, κ-, and δ- receptors and dopamine receptors D1 and D2 at sub-effective doses—doses confirmed not to influence feeding behavior when given alone. This experimental design allowed us to explore their interactive effects with kisspeptin without confounding autonomous action.

Following infusions, chicks were immediately returned to their designated boxes, where they were provided continuous ad libitum access to water and feed. Food intake was quantified within a two-hour time frame post-infusion. To control for inter-individual differences in body size, all feed intake data were normalized to the chick’s body weight and expressed as relative intake (g feed/kg body weight). To eliminate learning or habituation biases, each chick was subjected to a single treatment session. Injection site accuracy was verified post-mortem by histological assessment of frozen brain sections stained with Evans blue dye, confirming precise delivery to the targeted lateral ventricle. Data from all 308 injected animals were initially collected; however, final analysis was restricted to data from 286 animals with histologically confirmed correct injection placement. The analysis was restricted to data obtained from animals with confirmed correct injection placement.

### Statistical analysis

Statistical evaluation of cumulative food intake data, normalized to body weight, was conducted using two-way repeated-measures analysis of variance (ANOVA) to assess the effects of treatment over time. Where significant main effects or interactions were identified, pairwise group comparisons were performed using the Tukey–Kramer post hoc test. Statistical significance was defined as *P* < 0.05. Data are presented as mean ± standard error of the mean (SEM).

## Results

### Effect of kisspeptin on food consumption

**Fig 1:**
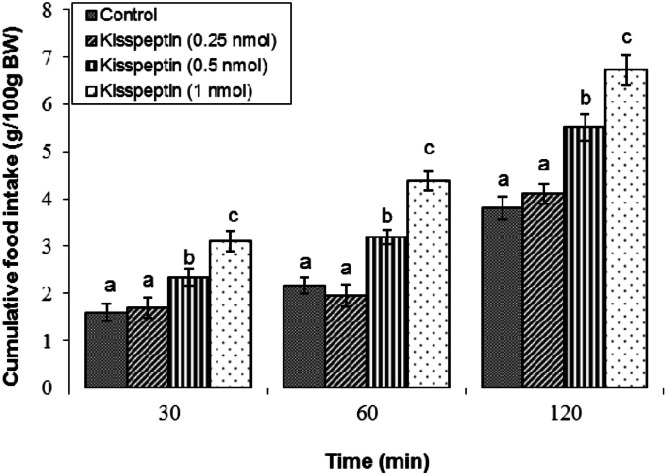
Cumulative food intake in broilers following central infusion of vehicle and kisspeptin (0.25, 0.5, and 1 nmol) (Control: *n* = 11; Kisspeptin 0.25 nmol: *n* = 8; Kisspeptin 0.5 nmol: *n* = 11; Kisspeptin 1 nmol: *n* = 11). Findings are presented as means ± SEM. Variations between groups are considered significant when represented using different alphabetic characters (a, b, c) (*P* < 0.05).

ICV administration of kisspeptin at a low dose (0.25 nmol) did not alter cumulative meal consumption in broilers compared to the controls across all time points assessed— within two hours following the infusion (*P* ≥ 0.05; Fig. 1). In contrast, higher doses of 0.5 nmol and 1 nmol kisspeptin resulted in an increase in feed consumption at each time interval measured (*P* < 0.05). Specifically, broilers receiving 1 nmol displayed the most pronounced hyperphagic response relative to controls.

### Effects of opioid receptor antagonists on kisspeptin -induced feeding

**Fig 2:**
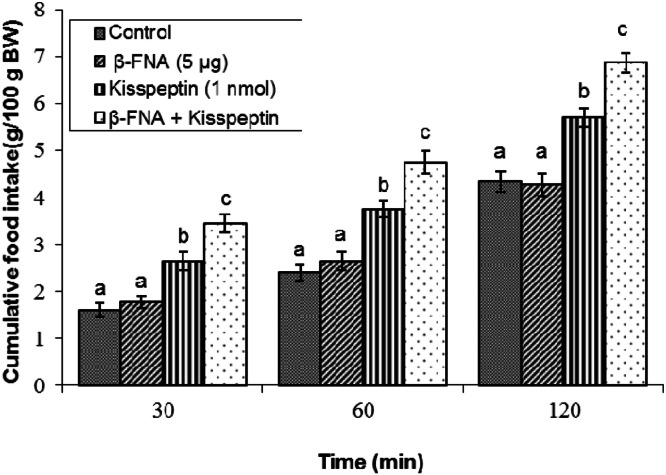
Cumulative food intake in broilers following central infusion of vehicle, β-FNA, kisspeptin, and their combination (Control: *n* = 10; β-FNA: *n* = 11; Kisspeptin: *n* = 11; β-FNA + Kisspeptin: *n* = 11). Findings are presented as means ± SEM. β-FNA is a selective mu receptor antagonist. Variations between groups are considered significant when represented using different alphabetic characters (a, b, c) (*P* < 0.05).

Effect of β-FNA on kisspeptin-induced feeding: Administration of β-FNA (5 μg) alone did not modify meal consumption compared to vehicle controls at any time point (*P* ≥ 0.05; Fig. 2). Kisspeptin alone enhanced cumulative feed intake during the two-hour period subsequent to the infusion (*P* < 0.05). Notably, co-infusion of β-FNA with kisspeptin increased this hyperphagic effect (*P* < 0.05).

**Fig 3:**
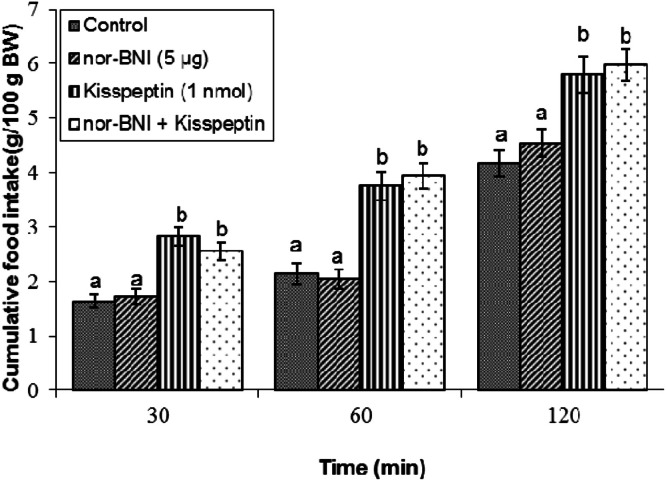
Cumulative food intake in broilers following central infusion of vehicle, nor-BNI, kisspeptin, and their combination (Control: *n* = 11; nor-BNI: *n* = 10; Kisspeptin: *n* = 9; nor-BNI + Kisspeptin: *n* = 11). Findings are presented as means ± SEM. Nor-BNI is a selective kappa receptor antagonist. Variations between groups are considered significant when represented using different alphabetic characters (a, b) (*P* < 0.05).

Effect of nor-BNI on kisspeptin-induced feeding: Administration of nor-BNI (5 μg) alone had no effect on meal consumption when compared to controls (*P* ≥ 0.05; Fig. 3). Kisspeptin increased feeding at all measured time points (*P* < 0.05). Co-infusion of nor-BNI with kisspeptin failed to modify the kisspeptin-induced orexigenic response (*P* ≥ 0.05).

**Fig 4:**
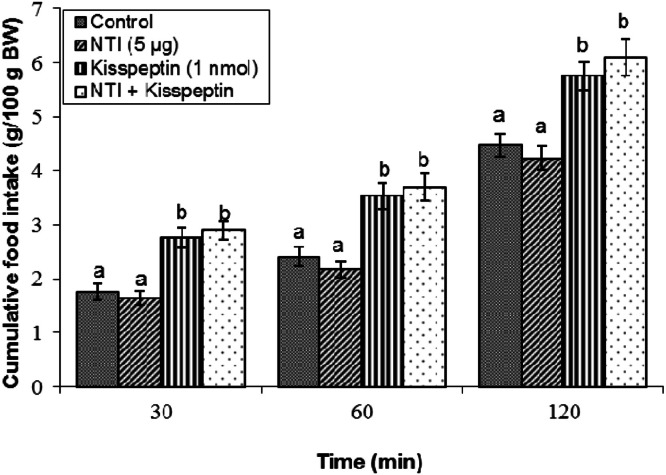
Cumulative food intake in broilers following central infusion of vehicle, NTI, kisspeptin, and their combination (Control: *n* = 10; NTI: *n* = 11; Kisspeptin: *n* = 9; NTI + Kisspeptin: *n* = 10). Findings are presented as means ± SEM. NTI is a selective delta receptor antagonist. Variations between groups are considered significant when represented using different alphabetic characters (a, b) (*P* < 0.05).

Effect of NTI on kisspeptin-induced feeding: NTI (5 μg) administered alone did not affect meal intake relative to vehicle-treated groups (*P* ≥ 0.05; Fig. 4). Kisspeptin increased cumulative food consumption within two hours following the infusion (*P* < 0.05). Co-infusion of NTI with kisspeptin did not alter the hyperphagia elicited by kisspeptin (*P* ≥ 0.05).

### Effects of dopaminergic agents on kisspeptin-induced feeding

**Fig 5:**
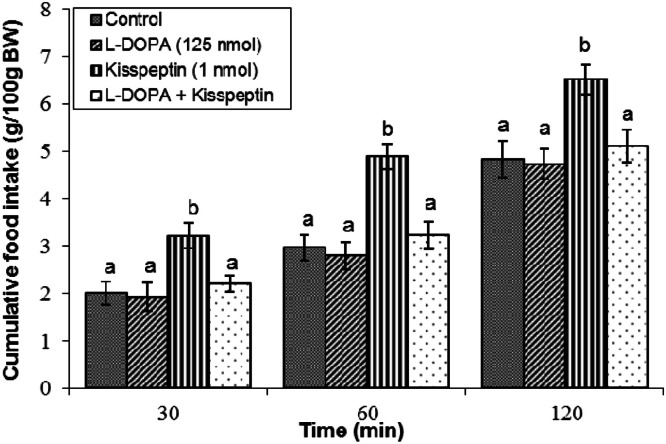
Cumulative food intake in broilers following central infusion of vehicle, l-DOPA, kisspeptin, and their combination (Control: *n* = 11; l-DOPA: *n* = 11; Kisspeptin: *n* = 9; l-DOPA + Kisspeptin: *n* = 8). Findings are presented as means ± SEM. l-DOPA is a dopamine precursor. Variations between groups are considered significant when represented using different alphabetic characters (a, b, c) (*P* < 0.05).

Effect of l-DOPA on kisspeptin-induced feeding: The dopamine precursor l-DOPA (125 nmol) was administered to determine if enhancement of dopamine synthesis modulates kisspeptin effects. l-DOPA alone did not influence feeding relative to vehicle across all observation times (*P* ≥ 0.05; Fig. 5). Kisspeptin elicited a stimulation of meal intake (*P* < 0.05), and co-infusion with l-DOPA mitigated the hyperphagic response (*P* < 0.05).

**Fig 6:**
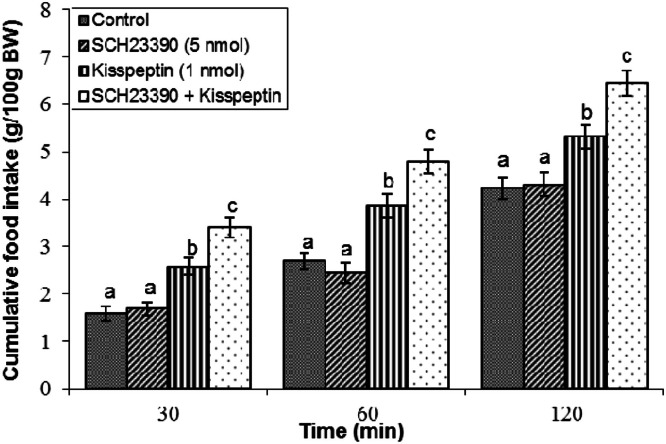
Cumulative food intake in broilers following central infusion of vehicle, SCH23390, kisspeptin, and their combination (Control: *n* = 11; SCH23390: *n* = 11; Kisspeptin: *n* = 11; SCH23390 + Kisspeptin: *n* = 10). Findings are presented as means ± SEM. SCH23390 is a selective D1 receptor antagonist. Variations between groups are considered significant when represented using different alphabetic characters (a, b, c) (*P* < 0.05).

Effect of SCH 23390 on kisspeptin-induced feeding: Administration of SCH23390 (5 nmol) alone did not modify meal consumption compared to vehicle controls at any time point (*P* ≥ 0.05; Fig. 6). Kisspeptin alone enhanced cumulative feed intake extending to two hours after administration (*P* < 0.05). Notably, co-infusion of SCH23390 with kisspeptin increased this hyperphagic effect (*P* < 0.05).

**Fig 7:**
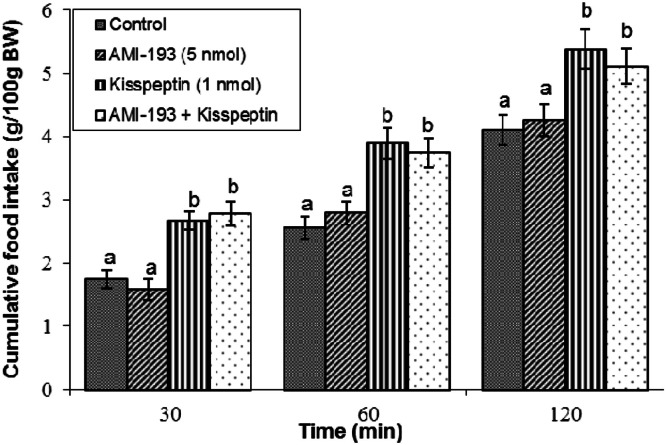
Cumulative food intake in broilers following central infusion of vehicle, AMI-193, kisspeptin, and their combination (Control: *n* = 10; AMI-193: *n* = 11; Kisspeptin: *n* = 10; AMI-193+ Kisspeptin: *n* = 8). Findings are presented as means ± SEM. AMI-193 is a selective D2 receptor antagonist. Variations between groups are considered significant when represented using different alphabetic characters (a, b) (*P* < 0.05).

Effect of AMI-193 on kisspeptin-induced feeding: AMI-193 (5 nmol) did not notably influence meal consumption when administered alone (*P* ≥ 0.05; Fig. 7). Kisspeptin administration continued to increase meal consumption relative to vehicle controls (*P* < 0.05). Importantly, concurrent administration of AMI-193 with kisspeptin did not alter the magnitude or temporal profile of kisspeptin-induced hyperphagia (*P* ≥ 0.05).

## Discussion

This investigation yields significant novel data regarding the role of central kisspeptin administration in regulating feeding behavior in meat-type chicks. We provided evidence that ICV infusion of kisspeptin significantly stimulates meal consumption dose-dependently. Specifically, doses of 0.5 and 1 nmol elicited robust hyperphagic responses, whereas a lower dose of 0.25 nmol had no remarkable effect. The 1 nmol dose increased cumulative intake by approximately 40 % compared to controls at the 120-min mark. These results indicate a dose threshold that must be reached to activate the orexigenic pathways modulated by kisspeptin in avian species.

Kisspeptin, originally identified as metastin and transcribed from the KiSS-1 gene, is widely recognized for its critical function in reproductive regulation, primarily via stimulation of hypothalamic GnRH neurons and potentially through direct action on gonadal cells, via its receptors (Masumi et al., 2022; Ohkura et al., 2009). The localization of kisspeptin receptors in the hypothalamus provides anatomical basis for its potential involvement in energy homeostasis and feeding control (Higo et al., 2016). Furthermore, Castellano et al. (2009) reported that energy storage within the body affects both the expression and functional role of hypothalamic KISS system, implying that kisspeptin may integrate nutritional status with reproductive and metabolic control. In mammals, however, central kisspeptin administration predominantly exerts anorexigenic effects by activating anorexigenic POMC neurons and inhibiting orexigenic NPY neurons, leading to decreased food intake (Fu and van den Pol, 2010). Notably, central kisspeptin administration in rodents often fails to alter food intake (Thompson et al., 2004), suggesting a limited or indirect role in feeding regulation .

In contrast, our findings in broiler chickens align more closely with recent avian studies that indicate an orexigenic effect of kisspeptin/metastin. For example, evidence in layer-type chickens suggests that metastin’s orexigenic effect may be mediated via NPY1 and GABAA receptors, which are key components in orexigenic signaling pathways (Kord et al., 2022). Additionally, evidence points to the mu-opioid receptor's engagement in chicks, further supporting kisspeptin’s stimulatory role in avian feeding behavior (Khan et al., 2009). This divergence between mammals and birds may reflect species-specific differences in hypothalamic circuitry and receptor expression.

A key aspect of our research was the examination of opioidergic and dopaminergic systems’ modulation of kisspeptin-induced feeding. To investigate this, we administered selective antagonists and agonists targeting μ-, κ-, and δ-opioid receptors and dopamine receptors D1 and D2 at sub-effective doses both individually and in combination with an effective dose of kisspeptin. This design allowed us to isolate their specific modulatory effects on kisspeptin signaling.

Our investigation into the opioidergic system revealed complex and unexpected interactions with kisspeptin-induced hyperphagia. While opioid receptors are G protein-coupled receptors with three subtypes (μ, κ, δ), their roles in feeding are species-dependent (Mahdavi et al., 2023a). In mammals, μ- and δ-opioid receptor activation generally promotes feeding, while receptor agonists have varied effects (Hadjimarkou et al., 2004; Kaneko et al., 2014). In contrast, avian studies have demonstrated that stimulation of κ- and δ-receptors tends to enhance food intake, whereas μ-opioid receptor activation has been proposed to exert an inhibitory influence on feeding (Bungo et al., 2005). Notably, investigations into the opioid receptor-mediated modulation of feeding responses elicited by diverse peptides and hormones in chickens have consistently identified the μ-opioid receptor as a pivotal mediator (Saffar et al., 2025; Zendehdel et al., 2016). These previous findings are congruent with the current study, wherein pharmacological blockade of μ-opioid receptors using β-FNA significantly augmented kisspeptin-induced hyperphagia. These data implicate mu receptor activation as a critical mediator of kisspeptin-induced hyperphagia. In addition to the results presented above, Our observations are in agreement with previous investigations revealing that antagonists of κ- and δ-opioid receptors does not significantly alter the hyperphagic response induced by kisspeptin administration, suggesting these receptor subtypes have a limited role in mediating this specific feeding behavior (Baghaeikia et al., 2022; Jaefari-Anari et al., 2018). This underscores the nuanced and species-specific complexity of opioid receptor functions within avian neuroendocrine circuits governing energy balance.

The dopaminergic system also fulfills a vital, albeit intricate, function in kisspeptin-induced feeding. Dopamine, a monoamine neurotransmitter with five receptor subtypes (D1-D5), is widely distinguished by its contribution to the reward, motivation, and appetite regulation (Klein et al., 2019). We observed that co-infusion of the dopamine precursor l-DOPA significantly mitigated kisspeptin-induced hyperphagia. This finding suggests that increased dopamine synthesis or signaling can counteract the orexigenic effects of kisspeptin. This aligns with previous avian studies showing that dopamine and l-DOPA can induce hypophagia in broilers (Zendehdel et al., 2014). Furthermore, selective antagonism of D1 dopamine receptors via SCH23390 markedly potentiated kisspeptin-induced hyperphagia, suggesting that activation of D1-like receptors exerts an inhibitory modulatory influence on the orexigenic pathways engaged by kisspeptin. These data implicate D1 receptor activation as a critical mediator of metastin-induced feeding stimulation. Given that D1-like receptors typically couple to stimulatory G-proteins to modulate intracellular signaling cascades (Kawahata et al., 2024), these findings imply a subtype-specific dopaminergic mechanism where D1 receptor activation functions as a regulatory "brake" on feeding stimulation. Notably, antagonist of D2 receptors did not exhibit a comparable effect in this experimental context, emphasizing distinct receptor subtype contributions within dopaminergic regulation of appetite. Consistent with these findings, prior research in chickens indicates that the modulatory roles of dopamine D1 and D2 receptors on food intake are influenced by strain and the specific neurochemical or hormonal system involved. For instance, in broiler chickens, oxytocinergic regulation of feeding predominantly involves D1-type receptors (Zarei et al., 2025b), whereas the Nociceptin/Orphanin FQ system exerts its effects through both D1- and D2-type receptors (Zendehdel et al., 2019). Furthermore, in laying hens, dopaminergic mediation of opioid’s effects on appetite engages D1 receptors (Zendehdel et al., 2016), while cannabinoidergic signaling appears to involve primarily D2 receptors (Khodadadi et al., 2017). These distinctions reflect complex, strain- and system-dependent receptor interactions within the dopaminergic circuitry that finely modulate meal consumption in response to diverse physiological signals and environmental cues.

The functional interplay between kisspeptin, dopaminergic, and opioidergic systems is not without precedent. These systems are known to interact within the hypothalamus, with documented crosstalk in the regulation of reproduction, such as the modulation of gonadotropin and prolactin release (Szawka et al., 2010; Ruka et al., 2013). The demonstration of their functional interaction in our study, within the domain of appetite control, extends this concept and underscores the multimodal nature of these neurochemical circuits. The shared anatomical substrates for these systems, particularly in hypothalamic nuclei critical for energy homeostasis (Hrabovszky, 2014; Romanov et al., 2017), provide a plausible framework for the integrated control of feeding behavior observed here.

These findings enhance our knowledge of the neurobiological mechanisms governing appetite in broiler chickens. The distinct orexigenic role of kisspeptin in broilers, contrasted with its typical anorexigenic effect in mammals, emphasizes the need for species-specific research in nutritional neuroscience. From a practical perspective, identifying these modulatory interactions could lead to novel strategies for optimizing feed efficiency and growth performance in poultry production, for example by developing dietary or management strategies that subtly modulate these neural pathways to promote optimal intake.

Nevertheless, some limitations should be noted. Our experimental framework is based on acute ICV administration, which may not fully represent chronic or peripheral regulatory influences on feeding behavior. Future research should focus on long-term interventions and elucidate molecular signaling pathways mediating these complex interactions. Additionally, identifying the precise neuronal circuitry and receptor co-localization patterns would deepen mechanistic understanding. Comparative studies across avian species and physiological states, such as fasting versus ad libitum feeding, will further refine the generalizability of these findings.

## Conclusion

In conclusion, central kisspeptin administration dose-dependently stimulates food intake in broiler chickens. The opioidergic μ-opioid and dopaminergic D1 receptor systems modulate this effect by exerting inhibitory control. These findings highlight sophisticated neurochemical integration in avian appetite control and offer promising targets for improving feeding efficiency and growth in poultry production. Future studies exploring chronic effects and molecular mechanisms will be crucial for translating these insights into practical applications.

## Funding

This research did not receive any specific grant from funding agencies in the public, commercial, or not-for-profit sectors.

## Declaration of generative AI and AI-assisted technologies in the writing process

During the preparation of this work the author(s) used Perplexity AI in order to identify and correct potential grammatical errors and improve the overall flow and readability of the manuscript. After using this tool, the author(s) reviewed and edited the content as needed and take(s) full responsibility for the content of the published article.

## CRediT authorship contribution statement

**Hamed Zarei:** Writing – review & editing, Project administration, Methodology, Formal analysis, Conceptualization. **Mohammadrasol Radmehr:** Writing – original draft, Investigation. **Alireza Mohtasham:** Writing – original draft, Investigation. **Mehran Mehdipour:** Writing – original draft, Methodology, Investigation. **Keyvan Hasani:** Writing – review & editing.

## Disclosures

The authors report no conflicts of interest.
